# Characteristics associated with progression to probable dementia with Lewy bodies in a cohort with very late-onset psychosis

**DOI:** 10.1017/S0033291724001922

**Published:** 2024-09

**Authors:** Lucy L Gibson, Christoph Mueller, Robert Stewart, Dag Aarsland

**Affiliations:** 1 King's College London, Institute of Psychiatry, Psychology and Neuroscience, London, UK; 2 South London and Maudsley NHS Foundation Trust, London, UK; 3 Centre for Age-Related Medicine, Stavanger University Hospital, Stavanger, Norway

**Keywords:** very late-onset psychosis, dementia, dementia with Lewy bodies

## Abstract

**Background:**

Very late-onset psychosis (VLOP) is associated with higher rates of dementia but the proportion who develop dementia with Lewy bodies (DLB) is unknown. We aimed to identify individuals with VLOP who develop dementia and DLB and characterize the risk factors for progression.

**Methods:**

Anonymized data were retrieved from electronic records for individuals with VLOP. Patients developing dementia after psychosis were identified, in addition to those with >2 core features of DLB at the time of dementia or DLB identified by a natural language processing application (NLP-DLB). Demographic factors, Health of the National Outcome Scale (HoNOS) and symptoms at index psychosis were explored as predictors of progression to dementia.

**Results:**

In 1425 patients with VLOP over 4.29 years (mean) follow up, 197 (13.8%) received a subsequent diagnosis of dementia. Of these, 24.4% (*n* = 48) had >2 core features of DLB and 6% (*n* = 12) had NLP-DLB. In cox proportional hazard models, older age and cognitive impairment at the time of psychosis were associated with increased risk of incident dementia. Visual hallucinations and 2+ core features of DLB at index psychosis were associated with increased risk of dementia with 2+ symptoms of DLB but not all-cause dementia. Two or more core features of DLB at index psychosis were associated with 81% specificity and 67% sensitivity for incident NLP-DLB.

**Conclusions:**

In patients with VLOP who develop dementia, core features of DLB are common. Visual hallucinations or two core features of DLB in VLOP should prompt clinicians to consider DLB and support further investigation.

## Introduction

Neuropsychiatric symptoms (NPS) are common manifestations of neurodegenerative dementias with major implications for patients and their caregivers. Psychotic symptoms in particular are associated with poorer quality of life and functional outcomes, accelerated cognitive decline, greater caregiver burden, earlier institutionalization, and reduced life expectancy (Aarsland, Larsen, Tandberg, & Laake, 2000; de Lau, Verbaan, van Rooden, Marinus, & van Hilten, 2014; Lee, McKeith, Mosimann, Ghosh-Nodyal, & Thomas, 2013; Schrag, Hovris, Morley, Quinn, & Jahanshahi, 2006). The profile of NPS differs across dementia subtypes; in clinical studies psychosis is commonest in dementia with Lewy bodies (DLB; 75% prevalence), followed by Parkinson's disease dementia (50%), Alzheimer's disease (AD; 40%), and frontotemporal dementia (10%) (Aarsland et al., 2007; Cummings et al., 2018; Fischer & Aguera-Ortiz, 2018; Ismail et al., 2022; Vik-Mo, Giil, Borda, Ballard, & Aarsland, 2020). Furthermore, up to 10% of people with mild cognitive impairment (MCI) report psychotic symptoms and in cognitively normal individuals psychotic symptoms are associated with an increased risk of incident cognitive impairment (Creese et al., 2020, 2023).

Psychotic disorders, such as schizophrenia, are also associated with increased risk of dementia, particularly where the onset of psychosis is >60 years in very late-onset psychosis (VLOP) (Almeida et al., 2019; Harvey et al., 1999; Heinrichs & Zakzanis, 1998; Howard, Rabins, Seeman, & Jeste, 2000; Korner, Lopez, Lauritzen, Andersen, & Kessing, 2009a, 2009b; Miniawi, Orgeta, & Stafford, 2022; Stafford et al., 2023; Yang, Sin Fai Lam, & Kane, 2021). The causal mechanisms underpinning this association are unknown but recent epidemiological and neuropathological evidence suggests that NPS, including psychosis, can be early manifestations of neurodegenerative disease indicating an ‘at-risk’ state for progression to dementia (Ismail et al., 2023; Krell-Roesch et al., 2019; McGirr et al., 2022; Ruthirakuhan, Ismail, Herrmann, Gallagher, & Lanctot, 2022; Wise, Rosenberg, Lyketsos, & Leoutsakos, 2019). The emergence of NPS after age of 50 and persisting for >6 months in advance of dementia is now termed mild behavioral impairment and a psychiatric-onset prodrome is also recognized in the recent research criteria for prodromal DLB (Donaghy et al., 2023; Ismail et al., 2016, 2017; McKeith et al., 2020).

With the increasing recognition of a psychiatric prodrome in neurodegenerative dementia (Utsumi, Fukatsu, Hara, Takamaru, & Yasumura, 2021), improved detection of psychosis as the index manifestation is critical for early diagnosis and intervention while the pathological and symptomatic burden is limited (Gibson, Abdelnour, Chong, Ballard, & Aarsland, 2023). This is of particular importance in DLB where the use of antipsychotic medications is associated with morbidity and mortality. However, although an increased incidence of dementia following VLOP is recognized, few studies have focused on DLB and it is not known what proportion develop DLB or other dementia subtypes (Stafford et al., 2023). NPS are more common in the prodromal stages of DLB than AD suggesting that rates of incident DLB might be increased in VLOP (Donaghy et al., 2022; McKeith et al., 2020; van de Beek et al., 2020; Wyman-Chick et al., 2022).

To date, the psychiatric-onset prodrome of DLB has not been well characterized and no clear distinguishing features have been identified to discriminate VLOP from psychosis in AD and DLB (Gunawardana, Matar, & Lewis, 2023; Van Assche et al., 2019). Although the presence of at least two core clinical features of DLB (parkinsonism, REM sleep behavior disorder [RBD], fluctuations, or visual hallucinations) can differentiate DLB from normal aging in the prodromal stages, this has not been explored widely across different clinical settings (McKeith et al., 2020; Wyman-Chick et al., 2022). In a cohort of patients in secondary mental health services with first onset of psychosis >60 years, we aimed to identify what proportion developed incident dementia and DLB. We further aimed to identify characteristics and risk factors at the time of VLOP associated with incident dementia and DLB.

## Method

### Sample

A retrospective cohort study was assembled from electronic clinical records data at the South London and Maudsley NHS Foundation Trust (SLaM) using the Clinical Record Interactive Search (CRIS). SLAM is one of the largest mental health providers in Europe, serving over 1.2 million residents across four South London boroughs (Lambeth, Southwark, Lewisham, and Croydon). CRIS was developed in 2008 to provide access to de-identified structured and free-text data in clinical records and a suite of natural language processing (NLP) algorithms, developed over this period, allows extraction of structured data from text fields (Cunningham, Tablan, Roberts, & Bontcheva, 2013; Fernandes et al., 2013; Perera et al., 2016), including for pharmacotherapy and a large number of signs or symptoms identified by clinicians (Cunningham et al., 2013). Details of NLP algorithm functionality and performance are contained in an open access catalogue (https://www.maudsleybrc.nihr.ac.uk/facilities/clinical-record-interactive-search-cris/cris-natural-language-processing/). CRIS received ethical approval as an anonymized database for secondary analysis for research purposes (Oxford REC C 23/SC/0257).

Patients were included in the study if they were >60 years of age when first diagnosed with a non-affective psychotic disorder in SLaM services between 1 January 2008 and 31 December 2021. Individuals aged at least 60 years were chosen to reflect the criteria for VLOP (Howard et al., 2000).

Date of diagnosis of non-affective psychosis served as the index date and patients were followed up until the date of dementia diagnosis (incident dementia), last face-to-face contact with secondary mental health services, death, or a censoring point on 31 December 2021. Patients were excluded if they had a diagnosis of dementia recorded within 6 months of the index date or if they had shorter than 6 months follow up after the index date. A cut-off of 6 months was chosen both to exclude contemporaneous dementia and psychotic disorder diagnoses and to reduce circular bias because, as described below, clinical symptoms were identified by NLP within 6 months of VLOP and dementia diagnosis, respectively. In online Supplementary Table 1 we also report the number of patients with VLOP who develop dementia 30 days after the index date, and separately, after 6 months, 1 year, or 2 years.

### Diagnosis

The International Statistical Classification of Diseases and Related Health Problems 10th Revision (ICD-10) criteria was used to classify cases of psychosis and dementia, both from diagnoses recorded in structured fields and in free text within clinical correspondence using an NLP algorithm.

### Late-onset psychosis

Diagnosis of non-affective psychosis was determined by diagnostic codes F20–29. Individuals with a first diagnosis of psychosis >60 years of age were included to reflect the definition of VLOP (Howard et al., 2000).

### Dementia

Diagnostic codes F00 (dementia in AD), F01 (vascular dementia), F02 (dementia in other diseases classified elsewhere), and F03 (unspecified dementia) were used to identify cases of dementia. DLB is often poorly coded by clinicians within CRIS and thus an NLP algorithm was used to code for this dementia subtype (Zixu Wang et al., 2020). Cases of DLB were identified with GATE NLP software to identify text strings of diagnostic statements of DLB (Zixu Wang et al., 2020). The diagnostic accuracy of this software in identifying DLB has previously been validated with a false-positive rate <5% (FitzGerald et al., 2019; Mueller et al., 2018; Perera et al., 2016). However, while NLP has high specificity for diagnosis of DLB in CRIS, a second validation study of NLP identified DLB in 2.0% of all cases of dementia in the wider cohort (200/10 159) (Mueller et al., 2018). This is markedly lower than the estimated prevalence of DLB in the community and suggests the sensitivity of NLP in identifying diagnoses of DLB may be low (Kane et al., 2018; Mueller et al., 2018). In order to mitigate this concern, we also report outcomes for patients with dementia and >2 core features of DLB recorded within 6 months of their dementia diagnosis and we also include patients identified as DLB by NLP in this group (irrespective of the number of core features). The core features were as per the McKeith criteria (McKeith et al., 2017) (I) ‘visual hallucinations’; (II) ‘fluctuations’; (III) ‘tremor’ or ‘bradykinesia’ as signs of parkinsonism; and (IV) ‘nightmares’ or ‘bad dreams’ as evidence of RBD and were identified in the free text of clinical correspondence using NLP applications.

### Covariates

Variables extracted included demographic factors (age at index date, gender, and ethnicity), psychotropic medication (antipsychotic and antidepressant) prescribed within 6 months of the index date, health status rated on the Health of the National Outcome Scale (HoNOS), and key clinical features identified with NLP at the time of psychosis diagnosis. The HoNOS is routinely administered in SLaM services and subscales for agitated behavior, hallucinations or delusions, cognitive problems, depressed mood, physical illness, or disability and activities of daily living were included in this study as symptoms which are commonly associated with DLB (Mueller et al., 2018). Each HoNOS item is scored from 0 (not present) to 4 (severe) and a score >2 was required to indicate disturbance in functioning in this domain. The exception was the cognitive problems item where a score of *>*1 was used to indicate impairment in order to capture individuals experiencing MCI. Previously developed NLP algorithms were used to identify key clinical signs and symptoms in the free text of correspondence. These included core features of DLB (visual hallucinations, fluctuations, RBD, parkinsonism) and physical symptoms such as falls and drowsiness. These symptoms were ascertained within 6 months either side of a psychosis diagnosis and were also separately ascertained 6 months either side of the dementia diagnosis.

### Statistical analysis

Baseline characteristics of the cohort were grouped according to incident all-cause dementia, dementia with 2+ core features of DLB and DLB-NLP only. Comparisons were made using χ^2^ tests for categorical variables or Fisher's exact tests where the count data were less than 5. Mann–Whitney tests were used to compare non-parametric continuous variables.

Cox proportional hazards analyses with the Breslow method were used to identify potential predictors for all-cause dementia and dementia with 2+ core features of DLB. A multivariate cox proportional hazard regression model for all predictor variables was not included for DLB due to the low number of DLB-NLP cases in the VLOP cohort. The hallucination and delusions item of the HoNOS was excluded from the models to avoid multicollinearity with visual hallucinations, a core feature of DLB. First, we report age, gender, and ethnicity-adjusted models (model 1) and significant predictors of these models are included in the multivariate model 2. Competing risk regression was also employed; the competing risk was death for all-cause dementia and other cause dementia for dementia with core features of DLB. For each variable the proportional hazard assumptions were assessed on the basis of Schoenfeld residuals and variables were included as time-varying if they were violated. Results are reported at a 5% significance level.

For each patient, the number of core features of DLB (visual hallucinations, fluctuations, parkinsonism, RBD) was identified around the time of psychosis. The association between the presence of 2+ core features of DLB at the time of psychosis and the risk of all-cause dementia, dementia with core features of DLB, and DLB-NLP, respectively, were assessed in cox proportional hazard regression models. Models were adjusted for age, sex, ethnicity, and cognition. The diagnostic accuracy of 2+ core features of DLB at the time of psychosis was assessed in sensitivity, specificity, and area under the (AUC) empirical receiver-operating characteristics (ROC) curves for DLB-NLP and dementia with core features of DLB. All analyses were performed using Stata version 18.0.

## Results

### Participants

During the 14-year study period between 2008 and 2021, 2204 patients received a first diagnosis of non-affective psychosis >60 years of age (see online Supplementary Table 1).

**Figure 1.**
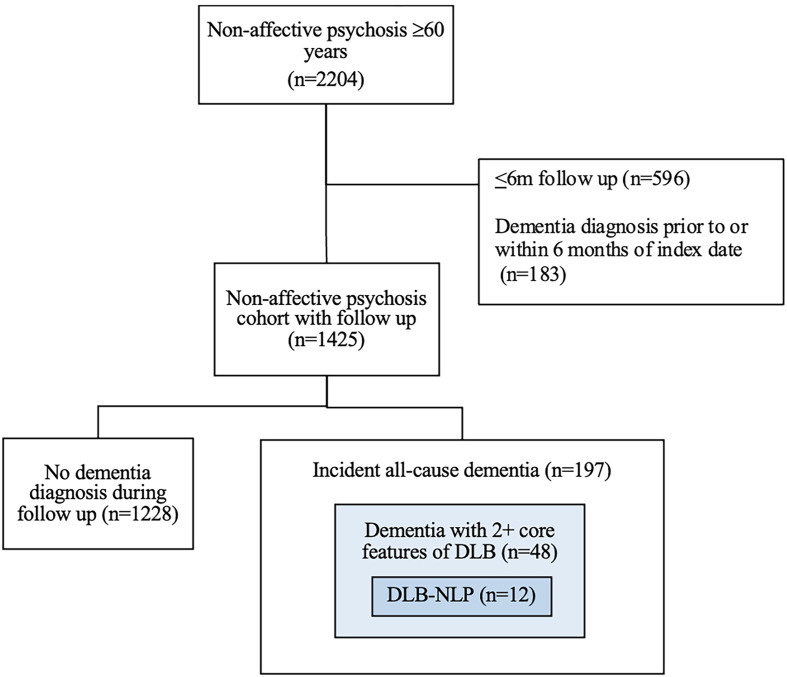
Flow chart of included patients.

Patients were excluded if a comorbid diagnosis of dementia was given prior to or within 6 months of psychosis index date (*n* = 183) or if they had <6 months of follow up from the index date (*n* = 596). The final cohort consisted of 1425 individuals with late-onset non-affective psychosis with mean follow up 4.29 years (3.27 s.d., range 0.5–14.0 years) (Fig. 1).

### Baseline characteristics of the late-onset psychosis cohort

In 197 (13.8%) cases, new onset dementia was diagnosed >6 months after the onset of psychosis. One-quarter (24.4%, *n* = 48) of those who developed dementia had at least two core features of DLB and 6.1% (*n* = 12) were identified by NLP as having DLB. Patients with incident dementia were older at first psychosis than those who did not develop dementia (*U* = −7.7, *p* < 0.001) but there was no significant difference in age of index psychosis for patients who developed DLB-NLP or other causes of dementia (*U* = 1.01, *p* = 0.32). The mean time to incident dementia was shorter for DLB-NLP than other causes dementia (*U* = 2.16, *p* = 0.03). The summary demographic and clinical features of the cohort are presented in Table 1.

**Table 1.** Baseline demographic and clinical features at the time of psychosis diagnosis for the whole VLOP cohort, patients who developed dementia, patients who had 2+ core features of DLB at the time of dementia diagnosis, and patients with DLB-NLP

	Total cohort	All-cause dementia^a^	Dementia + 2 core symptoms^b^	DLB-NLP^c^
Number of patients (%)	1425	197 (13.8)	48 (3.4)	12 (0.84)
Mean time in years from index psychosis to census (dementia/death/last clinical contact) (s.d.)^†^	4.29 (3.27)	3.93 (3.07)	3.74 (2.97)	2.51 (2.63) *
Time from diagnosis to death in years (s.d.)^†^	4.85 (3.08)	6.31 (3.12)**	5.50 (2.48)	5.77 (2.97)
Demographic				
Mean age at diagnosis (s.d.)^†^	72.3 (8.3)	76.3 (7.7)**	75.0 (7.7)	73.7 (8.1)
Female (%)	874 (61.3)	133 (67.5)	32 (66.7)	10 (83.3)^§^
Non-white ethnicity (%)	568 (41.6)	93 (47.2)	23 (47.9)	6 (50)
Pharmacotherapy (%)				
Antipsychotic use^#^	1134 (79.6)	161 (81.7)	38 (79.2)	9 (75.0)
Antidepressant use^#^	454 (31.9)	74 (37.6)	19 (40.0)	4 (33.3)^§^
HoNOS mental and physical health problems (%)				
Agitated behavior^+^	343 (24.1)	41 (20.8)	13 (27.1)	4 (33.3)^§^
Hallucinations or delusions^+^	910 (64.3)	146 (74.5)**	41 (87.2)*	11 (91.7)^§^
Depressed mood^+^	236 (16.6)	31 (15.8)	9 (19.2)	3 (25)^§^
Physical illness or disability^+^	604 (42.6)	84 (42.9)	22 (46.8)	7 (58.3)
Cognitive problems^^^	665 (47.1)	121 (62.1)**	31 (66.0)	9 (75)^§^
Mean score in cognitive problems item (s.d.)^†^	0.74 (0.94)	0.92 (0.94)**	0.98 (0.85)	1.08 (0.79)
ADLs	505 (37.8)	65 (33.3)	15 (31.9)	3 (25)^§^
Core symptoms of DLB (%)				
Visual hallucinations	343 (24.1)	52 (26.4)	22 (45.8)**	11 (91.7)^§^**
RBD (bad dreams or nightmares)	76 (5.3)	14 (7.1)	5 (10.4)	4 (33.3)^§^*
Parkinsonism (tremor or bradykinesia)	277 (19.4)	37 (18.9)	14 (29.2)*	3 (25)^§^
Fluctuations	373 (26.2)	59 (30.0)	17 (35.4)	4 (33.3)^§^
2 + core features at psychosis onset	274 (19.2)	39 (19.8)	16 (33.3)**	8 (66.7)^§^**
Mean number of core features (s.d.)^†^	0.75 (0.92)	0.82 (0.93)	1.21 (0.97) **	1.83 (0.94) **
Other symptoms (%)				
Falls	551 (38.7)	89 (45.2)*	24 (50)	5 (41.7)
Drowsiness	366 (25.7)	53 (26.9)	21 (43.8)**	6 (50)

^a^Comparison of all-cause dementia *v.* no dementia; ^b^dementia with 2+ core features of DLB *v.* dementia with <2 core features of DLB; ^c^DLB-NLP *v.* other cause dementia. ^†^Mann–Whitney test used to compare all non-parametric continuous data. In all remaining categorical comparisons χ^2^ test used unless the count was <5 denoted by ^§^where Fisher's exact test was used. ^#^Medication prescription in the 6 months before or after psychosis diagnosis as proxy for prevalent use at the time of index date; ^+^frequency of patients scored as experiencing problems >2 in that domain; ^^^frequency of patients scoring >1 in this item. **Significant at the 1% level, *p* < 0.01, *significant at the 5% level *p* < 0.05.

In patients with VLOP who developed dementia, the most common index diagnosis was schizophrenia (F20: 39%, *n* = 76), followed by delusional disorder (F22: 36%, *n* = 71), acute psychotic disorder (F23: 9%, *n* = 18), nonorganic psychosis (F29: 9%, *n* = 17), and schizoaffective disorder (F25: 3%, *n* = 6).

Cognitive problems (scoring >1 in the HoNOS cognitive item) were present in 47.1% of the total cohort at baseline (*n* = 665), and patients who subsequently developed incident dementia had greater frequency of cognitive impairment at baseline than those who did not (all-cause dementia 62.1% *v*. no dementia 44.6%, χ^2^ = 20.4, *p* < 0.001) with significantly higher mean scores in the HoNOS cognitive items. However, there was no significant difference in frequency of cognitive impairment at psychosis onset in patients with other causes of dementia and 2+ core features of DLB (2+ core features 66.0% *v*. other cause dementia 60.8%, χ^2^ = 0.40, *p* = 0.53) or DLB-NLP (DLB-NLP 75% *v*. other cause dementia 61.2%, Fisher's exact 0.54) (see Table 1).

Visual hallucinations were present in almost a quarter of the total cohort at diagnosis of VLOP (*n* = 343; 24.1%) and, at the time of psychosis, there was no difference in frequency of VH in patients who did or did not develop dementia (all-cause dementia 26.4% *v*. no dementia 23.8%, χ^2^ = 0.68, *p* = 0.41) (see Table 1). However, VH were significantly more common at the time of psychosis in patients who later developed DLB-NLP or dementia with core features of DLB *v*. other causes of dementia (DLB-NLP 91.7% *v*. other cause dementia 22.2%, Fisher's exact *p* < 0.001; dementia with 2+ core features 45.8% *v*. other cause dementia 20.1%, χ^2^ = 12.3, *p* < 0.001) (see Table 1). Two or more core features of DLB were present at time of psychosis in 19.2% (*n* = 274) of the total cohort and were significantly more common at baseline in patients with dementia and core features of DLB (33% dementia with 2+ core features *v*. 15.4% other cause dementia, χ^2^ = 7.32, *p* = 0.007) or DLB-NLP (DLB-NLP 66.7% *v*. other cause dementia 16.8%, Fisher's exact *p* < 0.001) (Table 1).

Antipsychotic prescribing was high at time of diagnosis of psychosis (79.6%) and there was no difference in prescribing at the time of psychosis diagnosis associated with future incident dementia (Table 1). Furthermore, antipsychotic prescribing remained high in the 6 months after dementia diagnosis and the frequency of antipsychotic prescriptions was particularly high for DLB (all-cause dementia 62.4% [*n* = 123]; dementia with 2 core features of DLB 81.3% [*n* = 39], DLB-NLP 75% [*n* = 9]).

### Predictors of dementia and dementia with core features of DLB

**Figure 2.**
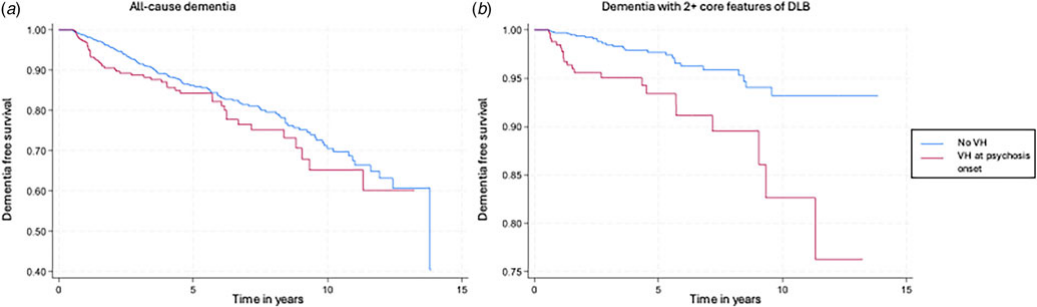
Kaplan Meier curves for probability of dementia-free survival with Cox proportional hazard models for patients with or without visual hallucinations at the time of index psychosis for (A) all-cause dementia and (B) dementia with 2+ core features of DLB.

In multivariate Cox proportional hazard models for both all-cause dementia and dementia with 2+ core features of DLB, older age was associated with increased risk of incident dementia. Cognitive impairment at baseline was also associated with increased risk of dementia (all-cause dementia HR = 3.43 [2.06–7.70], *p* < 0.001; dementia with 2+ core features of DLB HR = 1.98 [1.08–3.63], *p* = 0.028). However, in a fully adjusted model, visual hallucinations at baseline were associated with an increased risk of developing dementia with 2+ core features of DLB and at an accelerated rate (HR = 2.68 [1.42–5.08], *p* = 0.002) but not all-cause dementia (see Table 2 and Fig. 2). In subhazard ratio models adjusted for the competing risk of other causes of dementia, drowsiness and fluctuations at index psychosis were associated with an increased risk of incident dementia with 2+ core features of DLB (drowsiness SHR = 1.97 [1.04–3.75], *p* = 0.037; fluctuations SHR = 2.91 [1.05–8.02], *p* = 0.039) (Table 2).

**Table 2.** Multivariate cox proportional hazard regression models for all-cause dementia and dementia with 2 + core features of DLB

	All-cause dementia			Dementia with 2 + core symptoms of DLB		
	Model 1 (95% CI)	Model 2 (95% CI)	SHR (95% CI)	Model 1 (95% CI)	Model 2 (95% CI)	SHR (95% CI)
Demographics						
Age	1.07 [1.05–1.09] *p* < 0.001	1.07 [1.05–1.09] *p* < 0.001	1.05 [1.03–1.07] *p* < 0.001	1.05 [1.02–1.09] p = 0.003	1.05 [1.02–1.09] p = 0.003	1.04 [1.02–1.07] p = 0.003
Female	1.08 [0.80–1.46]	1.08 [0.80–1.47]	1.21 [0.90–1.65]	1.08 [0.59–1.98]		
Non-white	1.27 [0.96–1.69] p = 0.09	1.24 [0.93–1.65]	1.41 [1.06–1.87] p = 0.02	1.28 [0.72–2.26]		
HoNOS categories						
Agitation	0.83 [0.59–1.18]			1.19 [0.63–2.27]		
Physical illness	0.95 [0.72–1.27]^#^			1.16 [0.65–2.07]		
Cognitive problems	3.32 [1.99–5.54] *p* < 0.001^#^	3.14 [1.88–5.25] *p* < 0.001	3.43 [2.06–5.70] *p* < 0.001	2.08 [1.13–3.81] p = 0.02	2.01 [1.09–3.71] p = 0.026	1.98 [1.08–3.63] p = 0.028
Cognitive problems*time^^^	0.85 [0.77–0.94] p = 0.002	0.85 [0.77–0.94] p = 0.002	0.83 [0.75–0.92] *p* < 0.001			
Depressed mood	1.00 [0.68–1.48]			1.23 [0.60–2.55]		
Activities of daily living	0.83 [0.62–1.12]			0.79 [0.43–1.47]		
Core features						
Visual hallucinations	1.28 [0.93–1.77]			3.03 [1.70–5.39] *p* < 0.001	2.51 [1.34–4.70] p = 0.004	2.68 [1.42–5.08] p = 0.002
RBD	1.80 [1.04–3.12] p = 0.036	1.40 [0.80–2.46]	1.51 [0.89–1.80]	2.72 [1.07–6.93] p = 0.036	1.49 [0.57–3.89]	
Parkinsonism	0.85 [0.55–1.29]			1.81 [0.97–3.37] 0.06	1.19 [0.62–2.31]	
Fluctuations	1.63 [1.20–2.21] p = 0.002	1.35 [0.97–1.87]	1.27 [0.89–2.56]	4.80 [1.76–13.1] p = 0.002	2.73 [0.59–2.09] p = 0.06	2.91 [1.05–8.02] p = 0.039
Fluctuations* time^^^				0.76 [0.58–1.00] p = 0.05	0.76 [0.58–1.00] p = 0.05	0.75 [0.55–1.03] p = 0.07
Other symptoms						
Falls	1.48 [1.11–1.98] p = 0.008	1.26 [0.93–1.87]	1.11 [0.82–1.50]	1.88 [1056–3.36] p = 0.03	1.19 [0.62–2.31]	
Drowsiness	1.08 [0.79–1.49]			2.28 [1.29–4.05] p = 0.005	1.75 [0.94–3.28]	1.97 [1.04–3.75] p = 0.037
Pharmacotherapy						
Antipsychotic use	1.30 [0.90–1.87]			1.08 [0.54–2.18]		
Antidepressant use	1.51 [1.13–2.03] p = 0.005	1.33 [0.99–1.80]	1.32 [0.97–1.79]	1.59 [0.89–2.85]		

Model 1 adjusted for age, gender, and ethnicity. Model 2 adjusted for age, gender, ethnicity and significant or borderline predictors from model 1. SHR: subhazard ratio model 2 adjusted for competing risk of death for all-cause dementia and other cause dementia for dementia with core features of DLB. ^#^Stratified by ethnicity. ^^^Time-variable interactions describing how the hazard in the variable above changes per year.

In *n* = 3 (6.3%) of cases of dementia with core features of DLB, the core features associated with the dementia diagnosis may have been concurrent with core features within 6 months of the index psychosis. In these cases, the diagnosis of dementia was made less than one year after the index psychosis (range 0.58–0.88 years) and the core features were identified within 6 months of the psychosis and dementia diagnosis respectively. We ran sensitivity analyses excluding these cases with no overall change in the results.

### Core features of DLB

**Figure 3:**
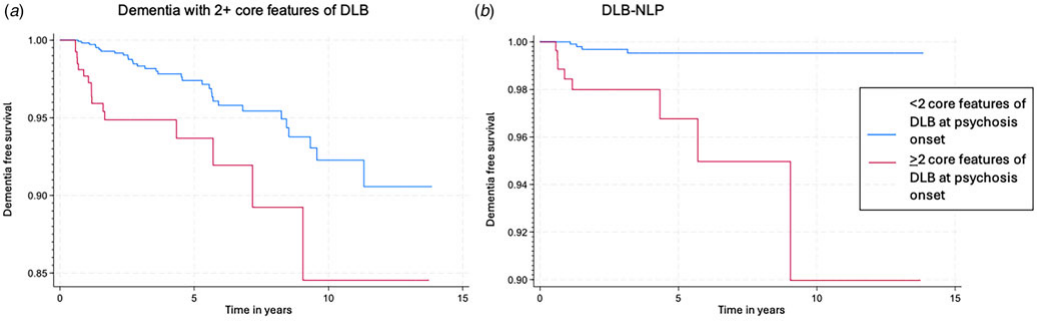
Kaplan Meier curves for probability of dementia-free survival with Cox proportional hazard models for patients with or without 2+ core features of DLB at the time of psychosis for (A) dementia with 2+ core features of DLB and (B) DLB-NLP.

In multivariate cox proportional hazard models adjusted for age, sex, ethnicity, and cognitive impairment, the presence of 2+ core features of DLB at index psychosis was not associated with increased risk of all-cause dementia (SHR = 1.25 [0.87–1.79], *p* = 0.23). However, the presence of 2+ core features at psychosis onset was associated with an increased risk of dementia with core features of DLB (SHR = 2.60 [1.44–4.69], *p* = 0.001) and DLB-NLP (SHR = 8.84 [2.68–29.1], *p* < 0.001) (see Fig. 3 and Table 3).

**Table 3.** Cox proportional hazards regression model for core features of DLB at the time of psychosis onset adjusted for age, sex, ethnicity, and cognition for all-cause dementia, dementia with 2 + core features of DLB, and DLB-NLP

	2 + core features of DLB at psychosis onset	
	HR	SHR
All-cause dementia	1.35 [0.95–1.92] *p* = 0.10	1.25 [0.87–1.79] *p* = 0.23
Dementia with 2 + core features of DLB	2.70 [1.47–4.96] *p* = 0.001	2.60 [1.44–4.69] *p* = 0.001
DLB-NLP	9.12 [2.72–30.6] *p* < 0.001	8.84 [2.68–29.1] *p* < 0.001

SHR: subhazard ratio, adjusted for competing risk of death for all-cause dementia and competing risk of other dementia for both dementia with 2 + core features of DLB and DLB-NLP.

The specificity of at least two core features at the time of psychosis onset for incident DLB-NLP was 81.2%, the sensitivity was 66.7% and the ROC area under the curve was 0.74 [95% CI 0.60–0.88]. The diagnostic accuracy of two core features at the time of psychosis was lower for dementia with 2+ core features of DLB with specificity of 81.3%, low sensitivity of 33.3%, and ROC AUC = 0.57 [95% CI 0.50–0.64].

## Discussion

In this longitudinal cohort with VLOP in a secondary mental health service, incident dementia was diagnosed in 13.8% of individuals over a mean duration of 4 years follow-up. Almost one-quarter (24.4%) had at least two core features of DLB at the time of dementia but only 6% of dementia cases were identified as DLB by NLP. Older age and cognitive impairment at psychosis onset were predictive of incident dementia, irrespective of the subtype. In addition, visual hallucinations or 2+ core features of DLB at the time of psychosis onset were associated with increased risk of dementia with core features of DLB but not all-cause dementia. Drowsiness and fluctuations at the time of psychosis was also associated with increased risk of developing dementia with core features of DLB when the competing risk of other causes of dementia was accounted for.

In clinical populations, DLB is estimated to comprise up to 7.5% of all dementia cases, while in community-based populations DLB is diagnosed in almost 5% of dementia cases (Kane et al., 2018; Vann Jones & O'Brien, 2014). However, a previous study in the wider healthcare setting from which our cohort was derived only identified DLB in 2% of all dementia cases (Mueller et al., 2018), a prevalence three times lower than we found in this VLOP cohort. Furthermore, we found almost one in four dementia cases had at least two core features of DLB and may represent possible DLB cases, an estimate closer to the frequency of Lewy body disease reported in post-mortem and biomarker studies (McAleese et al., 2021; Quadalti et al., 2023). Collectively, this suggests DLB may be disproportionately represented in individuals with VLOP who develop dementia (Mellergaard, Waldemar, Vogel, & Frederiksen, 2023; Vik-Mo et al., 2020; Wyman-Chick et al., 2022).

Psychosis is a common early clinical manifestation of DLB and visual hallucinations have been identified up to 5 years prior to diagnosis and in almost 25% 2 years prior to diagnosis (Fei et al., 2022; Wyman-Chick et al., 2022). However, while VLOP with significant functional impairment is increasingly recognized as a prodromal presentation of DLB, distinct from the construct of mild behavioral impairment, the typical clinical phenotype of psychiatric-onset prodromal DLB has yet to be characterized (Fei et al., 2022; Gunawardana et al., 2023; Kanemoto et al., 2022; McKeith et al., 2020; Urso et al., 2022). Studies have suggested up to one-third of patients with VLOP may have neurodegenerative changes but this is likely an overestimate because the sample sizes were small and only those with signs suspicious of AD or DLB underwent testing for indicative biomarkers (Kanemoto et al., 2022; Nagao et al., 2014; Satake et al., 2023).

Early diagnosis of DLB is critical to improve outcomes for patients and their caregivers and manage the prognostic and pharmacological implications of the disorder (Mueller, Ballard, Corbett, & Aarsland, 2017). Antipsychotics in particular can cause severe sensitivity reactions in DLB and were prescribed in 82% of this cohort with VLOP who developed dementia. However, to date, psychiatric-onset DLB has been difficult to differentiate from VLOP on the basis of symptoms alone; furthermore, although cognitive impairment at baseline is associated with incident dementia, accurate cognitive assessment is challenging in the context of severe psychotic symptoms (Gunawardana et al., 2023; McKeith et al., 2020; Van Assche et al., 2019). It is increasingly recognized that core features can differentiate DLB in its prodromal stages, even in the absence of cognitive impairment (McKeith et al., 2020; Wyman-Chick et al., 2022). Indeed, in our cohort with VLOP, the presence of at least two core clinical features or visual hallucinations at the time of psychosis onset was associated with both an increased risk of, and shorter time to, dementia with two core features of DLB. Visual hallucinations have also been associated with faster progression to dementia in a MCI cohort (Hamilton et al., 2021) suggesting visual hallucinations may be a key early indicator of prospective DLB. The presence of two core features of DLB at the time of psychosis was associated with specificity over 80% for DLB-NLP and the presence of these symptoms in VLOP should lead to a high index of suspicion, although the lower sensitivity highlights that not all future cases of DLB present with core features at the time of psychosis onset. The heterogeneity in the clinical presentation of DLB in the prodromal stages emphasizes the need for accurate, non-invasive biomarkers to support diagnosis and inform patient care (Gibson et al., 2023).

The longitudinal follow up of a large cohort of patients with VLOP is a major strength of this study, giving insight into the clinical course and evolution of symptoms while avoiding selection bias. The use of NLP applications allowed identification of individuals with DLB where few previous studies have discriminated dementia subtypes due to the underuse of diagnostic codes for specific dementias and the common misclassification of dementia subtypes in population-based health records (Butler, Kowall, Lawler, Michael Gaziano, & Driver, 2012; Rizzuto et al., 2018; Stafford et al., 2023). However, as we have discussed, NLP likely underestimates the number of cases of DLB in the cohort, particularly given the low sensitivity of diagnoses of DLB in clinical practice, with a high proportion of missed and misdiagnoses, in addition to misclassifications common in electronic health record data (Galvin et al., 2010; Kane et al., 2018). In order to address this concern we also identified all cases with dementia with at least two core features of DLB within 6 months of dementia diagnosis. However, the use of NLP to identify core features of DLB at the time of dementia diagnosis is not equivalent to a clinical diagnosis of DLB and requires validation. While we highlight risk factors at the time of index psychosis which are associated with incident dementia with features of DLB, further studies with more robust clinical or neuropathological confirmation of DLB are needed.

Other limitations associated with the use of routine electronic health record data also need to be considered. While NLP is a valuable tool for analysis in large datasets, it relies on the accuracy and completeness of the clinical information provided which is potentially highly variable and clinician-dependent with risk of underreporting of relevant clinical features (Perera et al., 2016). The current study relates to the timing between the diagnosis of psychotic disorder and dementia rather than the onset of symptoms and patient, caregiver, and service provider factors are associated with delays and inaccuracies in diagnosis (Bradford, Kunik, Schulz, Williams, & Singh, 2009). Furthermore, the psychotic symptoms of patients in this cohort met a threshold of persistence and severity to receive a diagnosis of psychosis in secondary care which limits the generalizability across all psychotic prodromal symptoms. In addition, assessments at the time of dementia diagnosis are likely to be biased by the history of symptoms reported by the individual such that presence of visual hallucinations is more likely to be explored if they have previously been reported. Finally, there was some circularity in the identification of the cohort of dementia with core features of DLB being made on the basis of core features within 6 months of the dementia diagnosis which in three (6.3%) cases occurred less than one year after the onset of psychosis (range 0.58–0.88 years). In these cases, core features occurring up to 6 months after the onset of psychosis may have overlapped with core features at the time of dementia diagnosis. However, sensitivity analyses excluding these cases showed no change in the overall significance of results.

Our findings suggest that DLB may be overrepresented where individuals with VLOP develop incident dementia but the causal nature of this relationship is not clear. Several studies have noted the risk of dementia is greatest in individuals with the shortest duration of psychosis, less than one year after VLOP, which suggests these psychotic symptoms may be an early manifestation of neurodegenerative disease (Almeida et al., 2019; Stafford et al., 2023). In further support of this, neuropathological change was more commonly found in individuals with late-onset schizophrenia, and early psychotic symptoms have been associated with both the greatest risk of progression to AD and fastest speed of decline (Ismail et al., 2023; Krell-Roesch et al., 2019; Nagao et al., 2014; Ruthirakuhan et al., 2022). However, the risk of incident dementia is also increased in individuals with early-onset psychosis and in those with prolonged follow up suggesting psychotic disorders could also represent a causal factor for dementia (Miniawi et al., 2022). The overlapping comorbidities and risk factors (cardiovascular disease, alcohol and substance misuse, smoking), use of antipsychotic medication, and physiological changes associated with psychotic disorder may increase the risk of dementia in this population. Moreover, a recent meta-analysis found individuals with cognitive decline in schizophrenia had similar rates of AD pathology to controls, suggesting there may be distinct mechanistic processes driving the late cognitive change associated with chronic psychotic illnesses and psychosis occurring in the prodromal stages of neurodegenerative disease (Wilson et al., 2024). Large, prospective studies with systematic evaluation of clinical features and biomarkers to support diagnosis and confirm evidence of early neuropathological changes are needed to address this question.

In summary, some patients with VLOP develop incident dementia and in those that do, core features of DLB are common. Visual hallucinations and core features of DLB at the time of psychosis onset should raise the index of suspicion for DLB and prompt further investigation. This is important both to guide the pharmacological management and to facilitate early diagnosis of DLB in individuals with high-risk features.

## Supporting information

Gibson et al. supplementary material Gibson et al. supplementary material

## Supplementary material

The supplementary material for this article can be found at https://doi.org/10.1017/S0033291724001922.
