# Supplementary material for: Characteristics associated with progression to probable dementia with Lewy bodies in a cohort with very late-onset psychosis
Source: Psychol Med. 2024 Sep 26;54(12):3479–88. doi: 10.1017/S0033291724001922 (PMC11496220; doi:10.1017/S0033291724001922)
Supplement: Gibson et al. supplementary material [file S0033291724001922sup001.docx]

| Time to census (dementia/death/last follow up) | All patients | All-cause dementia | Dementia with 2+ core features of DLB | DLB-NLP |
| --- | --- | --- | --- | --- |
| < 30 days | 2204 | 380 (17.2) | 107 (4.9) | 28 (1.3) |
| > 30 days | 1677 | 234 (14.0) | 67 (4.0) | 16 (1.0) |
| > 6 months | 1425 | 197 (13.8) | 48 (3.4) | 12 (0.8) |
| > 1 year | 1247 | 172 (13.8) | 40 (3.1) | 8 (0.6) |
| > 2 years | 980 | 128 (13.1) | 29 (3.0) | 4 (0.4) |
| > 5 years | 479 | 59 (12.3) | 16 (3.3) | 2 (0.4) |

Supplementary Table 1. Number of patients developing dementia, dementia with core features of DLB and DLB-NLP at different census intervals.
